# Ablation of NK Cell Function During Tumor Growth Favors Type 2-Associated Macrophages, Leading to Suppressed CTL Generation

**DOI:** 10.1080/10446670310001626580

**Published:** 2003

**Authors:** Anja B. Geldhof, Jo A. van Ginderachter, Yuanqing Liu, Wim Noël, Patrick de Baetselier

**Affiliations:** Laboratory of Cellular Immunology Free University of Brussels Flemish Interuniversity Institute for Biotechnology B-1640 St.-Genesius Rode Belgium

## Abstract

Several reports describe regulatory interactions between NK cells and CTLs. We addressed the issue of NK participation in the early anti-tumor defense by inoculating α-ASGM-1 treated mice with BW-Sp3 T lymphoma. Rejection of BW-Sp3 depends on strong CTL responses. Our results demonstrated that (i) NK cells are a prerequisite for efficient CTL generation and (ii) the absence of NK cells favors the outgrowth of alternatively activated macrophages that can suppress CTL restimulation. *In vitro* studies demonstrate that in splenic cultures from NK-deficient, tumor-bearing mice, the presence of alternatively activated macrophages correlates with a lack of Type 1 cytokines, while the production of Type 2 cytokines is promoted. Provision of the Type 1 cytokine, IFN-γ can boost overall CTL activity but does not revert the dominance of arginase producing adherent cells in the NK-deficient CTL cultures. The role of NK effector functions in the efficient switch of the immune system towards Type 1 activation was evaluated in cytotoxicity assays. The results indicate that the accessory function of NK can depend at least partially on their ability to preferentially engage arginase-producing cells, suggesting that NK/macrophage lytic interactions might be involved in the switch from Type 2 to Type 1-dependent immune responses.


*Clinical & Developmental Immunology*, June–December 2003 Vol. 10 (2–4), pp. 71–81

Taylor & Francis healthsciences

### Ablation of NK Cell Function During Tumor Growth Favors Type 2-Associated Macrophages, Leading to Suppressed CTL Generation

ANJA B. GELDHOF*, JO A. van GINDERACHTER, YUANQING LIU, WIM NOËL and PATRICK de BAETSELIER

*Laboratory of Cellular Immunology, Free University of Brussels, Flemish Interuniversity Institute for Biotechnology, B-1640 St.-Genesius Rode, Belgium*

Several reports describe regulatory interactions between NK cells and CTLs. We addressed the issue of NK participation in the early anti-tumor defense by inoculating α-ASGM-1 treated mice with BW-Sp3 T lymphoma. Rejection of BW-Sp3 depends on strong CTL responses. Our results demonstrated that (i) NK cells are a prerequisite for efficient CTL generation and (ii) the absence of NK cells favors the outgrowth of alternatively activated macrophages that can suppress CTL restimulation. *In vitro* studies demonstrate that in splenic cultures from NK-deficient, tumor-bearing mice, the presence of alternatively activated macrophages correlates with a lack of Type 1 cytokines, while the production of Type 2 cytokines is promoted. Provision of the Type 1 cytokine, IFN-γ can boost overall CTL activity but does not revert the dominance of arginase producing adherent cells in the NK-deficient CTL cultures. The role of NK effector functions in the efficient switch of the immune system towards Type 1 activation was evaluated in cytotoxicity assays. The results indicate that the accessory function of NK can depend at least partially on their ability to preferentially engage arginase-producing cells, suggesting that NK/macrophage lytic interactions might be involved in the switch from Type 2 to Type 1-dependent immune responses.

*Keywords*: Alternatively activated macrophages; CTL; Depletion; NK

### INTRODUCTION

The contribution of NK cells in the regulation of CTL activity has been documented in a number of experimental models, including xenospecific CTL generation (Smyth *et al.*, 1998; Smyth and Kelly, 1999), induction of influenza virus-specific CTLs (Kos and Engleman, 1996) and priming of tumor-specific CTLs (Kurosawa *et al.*, 1995; Terao *et al.*, 1996). Overall, in these studies the mechanisms underlying NK-mediated regulation of CTL activity were not exactly defined and remain controversial. Indeed, Smyth *et al.*, demonstrated that, though $CD4^+$ T cells are critical for a mouse anti-human xenospecific $CD8^+$ CTL response, $NK1.1^+$ cells play an IFN-γ-dependent accessory role in generating a maximal CTL response at the level of the lymph nodes (Smyth *et al.*, 1998; Smyth and Kelly, 1999). On the other hand, Kos and Engleman provided evidence that NK cells, but not NK-derived soluble factors, are strictly required for the generation of influenza virus-specific CTL following infection (Kos and Engleman, 1996). Finally, in the case of $CD4^+$-dependent priming of B16 tumor-specific CTLs, Terao *et al.* found that depletion of NK cells before B16-immunization abrogated tumorspecific CTL activity. Furthermore, they observed that NK cells had a promoting effect on priming of $CD4^+$ cells, but inhibited the priming of $CD8^+$ T cells (Terao *et al.*, 1996). Kurosawa *et al.* refined the latter study and observed that part of the anti-tumor CTL activity in NK-depleted mice was restored by i.p. injections of IL-2 and to a lower extent of IFN-γ (Kurosawa *et al.*, 1995).

Besides an immunoregulatory role of NK cells through cytokine production, these cells might also perform accessory functions through cell–cell contact-dependent interaction with antigen presenting cells (APC). Both positive (activating) (Warschkau and Kiderlen, 1999) and negative (lytic) (Gilbertson *et al.*, 1986; Djeu and Blanchard, 1988a,b; Chambers *et al.*, 1996; Geldhof *et al.*, 1998a; Carbone *et al.*, 1999; Parajuli *et al.*, 1999; Wilson *et al.*, 1999a,b; Spaggiari *et al.*, 2001) regulatory interactions have been substantiated. In the latter condition, several APC surface molecules were found to be possibly involved in the lytic interaction, either activating such as B7-1/B7-2 (Chambers *et al.*, 1996; Geldhof *et al.*, 1998a; Wilson *et al.*, 1999a,b),

*Corresponding author. Address: Department of Cellular Immunology, VIB-VUB, Pleinlaan 2, B-1050 Brussel, Belgium. Fax: 32-2-629-33-89. E-mail: ageldhof@cntnl.jnj.com

ISSN 1740-2522 print/ISSN 1740-2530 online © 2003 Taylor & Francis Ltd
DOI: 10.1080/10446670310001626580

CD40L (Carbone *et al.*, 1999), CD54 (Parajuli *et al.*, 1999), HLA (Spaggiari *et al.*, 2001), or inhibitory such as CD1 (Carbone *et al.*, 2000) and MHC class I molecules (Ferlazzo *et al.*, 2001). As such, one might assume that the functional result of the interaction is determined by the sum of the opposing signals. Moreover, the outcome of NK/APC interaction seems to depend on the cytokine milieu (Ferlazzo *et al.*, 2001; Spaggiari *et al.*, 2001). The implications of these NK/APC interactions for the adaptive immune response however remain speculative.

According to recent studies, different subsets of APC can develop, depending on the resident type 1/type 2 cytokine balance, namely classically activated APC (DC1/M1 or type 1-associated) versus alternatively activated APC (DC2/M2 or type 2-associated) and both are antagonistically regulated. DC1/M1 are the final targets and effectors of pro-inflammatory processes (Goerdt *et al.*, 1999; Pulendran *et al.*, 2001), while DC2/M2 appear to participate in anti-inflammatory processes, tolerance induction and wound healing and express a distinct set of molecules and receptors used in innate immunity (Stein *et al.*, 1992; Goerdt and Orfanos, 1999; MacDonald *et al.*, 2001). Both subtypes of APC can however exert inducing and/or downregulatory effects on adaptive immune responses.

In this study, we have analyzed the possible involvement of NK cells (via either cytokine production and/or cytolytic activity) in determining the cellular composition of APCs and evaluated the resulting effects on anti-tumor CTL responses. To this end the BW-Sp3 lymphoma model was adopted since rejection of BW-Sp3 depends on eliciting strong CTL responses (Van den Driessche *et al.*, 1994; Raes *et al.*, 1998) and consequently, this model is appropriate to examine the involvement of NK cells in early $CD8^+$-dependent immune reactions. To address the importance of NK cells for the early anti-tumor defense, α-ASGM-1-treated mice were challenged with BW-Sp3 cells. Use of α-ASGM-1 to deplete NK cells was considered a well-founded option, considering our and other available information (Beck *et al.*, 1982; Stitz *et al.*, 1986; Stout *et al.*, 1987). Our results indicate that NK cells are a prerequisite for efficient CTL generation and that absence of NK cells favors the outgrowth of a macrophage population that can be characterized by the dominant presence of $CD11b^+$/$GR\text{-}1^+$ and alternatively activated adherent cells. Subsequently, we show that these M2 cells, associated with NK depletion, suppress the restimulation of CTLs. Finally, preferential engagement of M2 by activated NK (LAK) cells reveal that cytolytic interactions might be involved in the regulatory function of NK cells to drive M1-dependent CTL responses.

### RESULTS AND DISCUSSION

### Tumor Progression is Favored by NK Cell-depletion

Previously, we have demonstrated that upon subcutaneous inoculation, BW-Sp3 T lymphoma cells form primary tumors that will either progress or regress (Fig. 1; Raes *et al.*, 1995). The immune effectors involved in this process reside primarily in the $CD8^+$ T cell population. Successful tumor rejection seems to depend on the generation of an efficient CTL response within 12 days after tumor challenge, a process that in our tumor model is mainly $CD4^+$ helper T cell independent (Van den Driessche *et al.*, 1994; Raes *et al.*, 1995, 1998). Indeed, depletion of $CD8^+$ T cells results in tumor progression in 100% of the recipients (Fig. 1). Furthermore, we have shown that all mice that have rejected the primary tumor challenge, will remain tumor-free upon rechallenge of BW-Sp3 cancer cells in the opposite flank (Fig. 1). These results indicate that subcutaneous inoculation of BW-Sp3 cells can result in the generation of an adaptive immune memory to BW-Sp3-dependent antigens and indicate that the BW-Sp3 tumor cell line is immunogenic. However, notwithstanding the above-established CTL-dependence of tumor rejection, also NK cells seem to have a decisive role in overcoming BW-Sp3 tumors. Indeed, inoculation of BW-Sp3 in NK-deficient mice will also prevent efficient clearance of the tumor cells as indicated by increased tumor growth rate, early death of most of the recipients (50% of the mice die within 19 days, Fig. 1A) and a significant increase in progressing tumors (from 66% in immunocompetent mice to 100% in NK-depleted counterparts, Fig. 1B). Before we pursued the importance of NK cells in early local anti-tumor immune responses, we verified that the antibody we used for depletion of NK cells in our AKR model, namely anti-ASGM-1, was uniquely targeting NK cells and did not eliminate BW-Sp3-specific CTL. Indeed, some reports suggest that α-ASGM-1 treatment might also reduce the pool of Ag-specific CTL, especially memory CTL raised during viral infections (Parker *et al.*, 1988; Slifka *et al.*, 2000). As expected, both the $DX5^+$ and $NKG2^+$ NK populations, which are almost exclusively $ASGM\text{-}1^+$, are severely reduced by α-ASGM-1 treatment (results not shown). However, α-ASGM-1-depletion did not significantly influence the number of antigen-specific $ASGM\text{-}1^+$/$CD8^+$ T cells, as indicated by FACS (results not shown) and by BW-Sp3-directed CTL assays. Indeed, α-ASGM-1-treatment of mice that had rejected a BW-Sp3 tumor challenge, did not influence the efficiency of CTL activity when spleens were tested 2 weeks after antibody treatment (Fig. 2). At this time point NK cells could have repopulated, but not BW-Sp3-specific memory CTL, since mice were absolutely tumor-free. This indicates that α-ASGM-1 depletion primordially affects NK cells, but not Ag-specific T cells. Thus, NK cells may be implicated in local antitumor responses against BW-Sp3.

### Depletion of NK Results in Enrichment of $CD11b^+$/$GR\text{-}1^+$ Cells in the Spleen

Studies from others (Apolloni *et al.*, 2000; Bronte *et al.*, 2000, 2001; Mazzoni *et al.*, 2002) and our laboratory

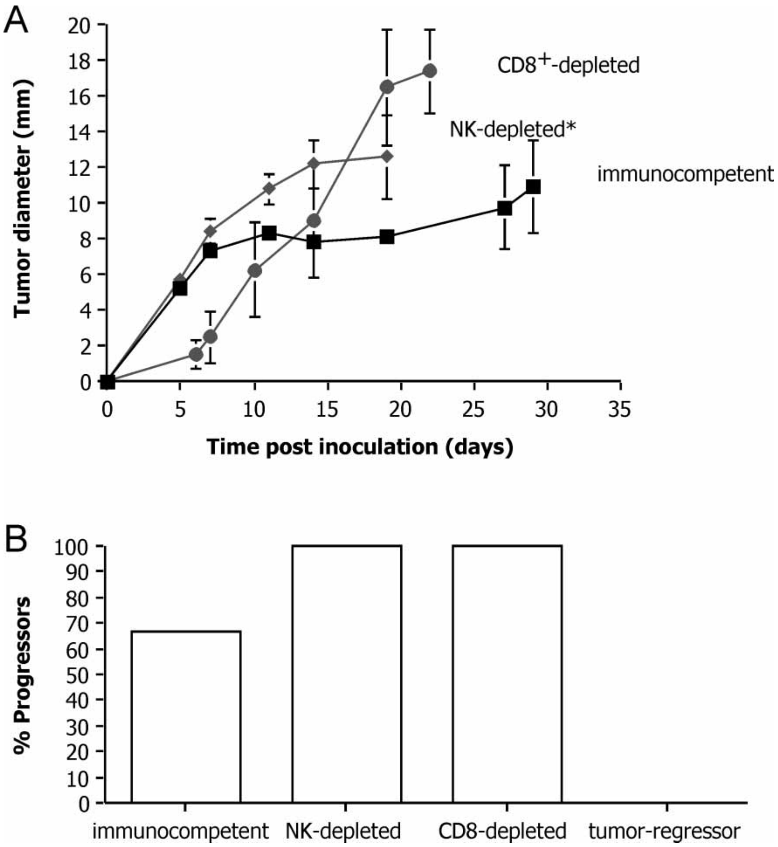

FIGURE 1 NK cells positively influence the efficient rejection of BW-Sp3 T cell lymphoma upon subcutaneous injection. Tumor cells ($2.10^6$) were injected subcutaneously in the right flank of immunocompetent or anti-ASGM-1-treated AKR mice. (A) tumor diameter was measured in function of time; results are represented as the mean tumor diameter (± s.e.m.) of (i) immunocompetent mice (■), (ii) NK-depleted mice (◆) or (iii) CD8-depleted mice (●) and (B) Percentage of mice with progressing tumors was plotted for naïve mice, NK-depleted mice, CD8-depleted mice and mice that have rejected the tumor load, after *de novo* subcutaneous tumor injection in the opposite flank. *Indicates the time point where 50% of the α-ASGM-1 depleted hosts prematurely succumbed to the tumor load.

(unpublished results) correlated advanced tumor progression with the emergence of a suppressive myeloid population at the level of the spleen. Bronte *et al.*, identified tumor-produced GM-CSF as the factor responsible for the recruitment of these GR-1$^+$/CD11b$^+$ immature myeloid progenitors to the spleen (Bronte *et al.*, 1999). Since BW cells do not produce GM-CSF (results not shown) this cannot explain their presence in our late-stage progressors. We have demonstrated in our earlier studies that NK cells can negatively influence the presence of APC (Geldhof *et al.*, 1998a), while other studies have provided additional evidence for pleiotropic NK/APC interactions (Gilbertson *et al.*, 1986; Djeu and Blanchard, 1988a,b; Chambers *et al.*, 1996; Carbone *et al.*, 1999; Parajuli *et al.*, 1999; Warschkau and Kiderlen, 1999; Wilson *et al.*, 1999a,b; Spaggiari *et al.*, 2001). Based on our observation that the absence of NK cells promotes tumor progression early during tumor growth, we wanted to test the effect of NK depletion on the distribution of myeloid cells in the tumor-challenged spleen.

To this end, we examined the distribution of myeloid surface markers in fresh spleens of immunocompetent and NK-depleted mice, 7 days after BW-Sp3 tumor inoculation. Based on the expression of CD11b and GR-1 (a mAb recognizing Ly-6G and Ly-6C) and the FSC/SSC profiles, we subdivided the spleen cells into five populations (Fig. 3i, iii). Globally, we obtained a moderate, but sustained increase in GR-1$^+$/CD11b$^+$ double positive cells, from 4.2% in immunocompetent-to 5.9% in NK-depleted hosts (Fig. 3). In late-stage progressors this increase is more substantial and can be 10-fold higher (Bronte and unpublished results). Notably, besides moderate increase in number, we could also detect shifts in surface phenotype. GR-1$^{high}$/CD11b$^{high}$/SSC$^{high}$ cells, comprising mainly neutrophils (Fig. 3i, iii; population A) express only marginal levels of F4/80 and CD11c. However, upon depletion of NK cells a new population appears that expresses high levels of F4/80 and thus acquires a macrophage phenotype (Fig. 3i). GR-1$^+$/CD11b$^+$/SSC$^{low}$ macrophages (population BE) are homogenous in both recipients for F4/80 (high) and CD11c (marginal) expression, though their number also tends to increase in NK-depleted tumor-bearing hosts. A second subpopulation of group B (population BD) has a higher SSC profile, though is comparable in F4/80 and CD11b expression levels to population BE. GR-1$^{low}$/CD11b$^+$ splenocytes, a mixed population containing DC

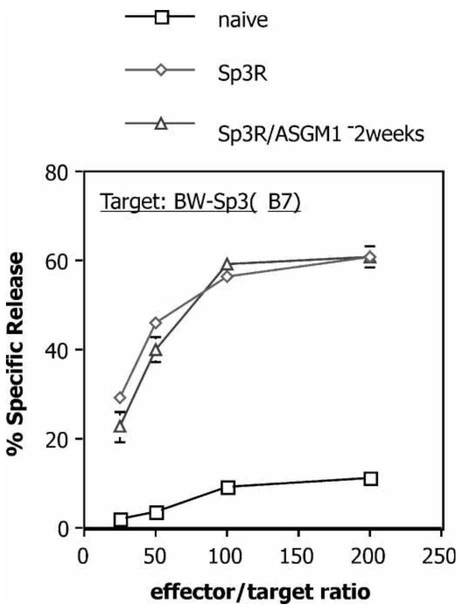

FIGURE 2 α-ASGM-1 treatment predominantly targets NK cells, but not activated T cells. Splenocytes from naïve mice (□), mice that rejected the local tumor load (◇), or mice that had rejected the BW-Sp3 tumor load and received one α-ASGM-1 treatment 2 weeks prior (Δ), were collected and were restimulated *in vitro* with irradiated BW-Sp3(B7-1) cancer cells. After 5 days, viable lymphocytes were collected. Cytolytic activity of effector CTL was measured in a 4 h $^{111}$In-release assay against BW-Sp3(B7-1) target cells at different Effector/Target ratios. Results are represented with SEM.

and other macrophages seems to be severely reduced in CD11c$^{high}$ (DC) cells upon depletion of NK cells, indicating that the proportion of a homogenous GR-1$^{low}$/CD11b$^{high}$/F4/80$^{high}$/CD11c$^{+}$ population increased. Altogether, in NK-depleted tumor-bearing mice, the splenic macrophage population increases in number as well as in F4/80 and CD11b expression level (Fig. 3ii, iv). Together with the results obtained in late tumor progressors, this may suggest that NK/myeloid innate interactions might be involved in shaping the adaptive BW-Sp3-directed immune response, deciding between tumor-regression or -progression.

### CTL Activity is Impaired in NK-depleted Mice and can be Partially Reconstituted by Removal of the Adherent Splenic Fraction

Since BW-Sp3 tumor rejection relies on both CD8$^{+}$ cytolytic T cells (Van den Driessche *et al.*, 1994; Raes *et al.*, 1995, 1998) and NK cells (see section "Introduction"), the role of NK cells in early antigen-specific CTL activity in tumor-bearing mice was investigated. Splenocytes of 7-days tumor-bearing immunocompetent and NK-depleted mice were restimulated with Sp3(B7-1) *in vitro* for 5 days and subsequently tested for their CTL activity. In addition, we also evaluated the functional importance of splenic myeloid cells for the efficiency of the CTL response (see section "Results and Discussion") by performing CTL restimulation in the absence or presence of this cell population. Already 7 days post tumor inoculation, immunocompetent mice generated significant levels of specific effector CTL's (15% lysis for $E/T = 100/1$), while on the contrary, NK cell depletion prevented the generation of CTL effector function (4.5% lysis for $E/T = 100/1$), barely rising above the lytic activity of restimulated naïve splenocytes (3% lysis) (Fig. 4). The inferior CTL activity of *in vitro* restimulated splenic cultures, derived from α-ASGM-1-treated mice, could be correlated with a lower percentage (12 vs. 8.5% CD8$^{+}$, Fig. 5A) and a lower level of activation (5% CD8$^{+}$CD69$^{+}$ vs. 2.5% CD8$^{+}$CD69$^{+}$) of CD8$^{+}$ T cells (Fig. 5A). The absence of NK cells does not reduce the number of CD4$^{+}$ splenocytes nor their activation status in the restimulated CTL cultures (Fig. 5A). Thus, NK cells play an accessory role in the generation of tumor-specific CTL.

Removing the splenic myeloid cells after 2 days of restimulation, and further culturing the non-adherent splenocytes for another 3 days, we saw an increase in the lytic activity of splenocytes from as well immunocompetent (from 15% in total to 31% in non-adherent splenocytes for $E/T = 100/1$) as NK-depleted (from 4.5% in total to 19.3% in non-adherent splenocytes for $E/T = 100/1$) 7 days BW-Sp3 tumor-bearing mice (Fig. 4). These results suggest that in both conditions, adherent myeloid cells can suppress the efficient amplification of CTL effectors, though the effect predominates in the NK-depleted condition, where CTL activity quadruplicates upon removal of the adherent population, as compared to the immunocompetent condition where target lysis only doubles (Fig. 4). Presuming that there is a correlation between suppression and presence of CD11b$^{+}$GR-1$^{+}$ cells (Bronte *et al.*, 1999, 2000, 2001; Apolloni *et al.*, 2000; Mazzoni *et al.*, 2002), we can corroborate the abovementioned findings by our FACS data. Indeed, the adherent fractions of both CTL cultures express significant levels of the CD11b$^{+}$GR-1$^{+}$ double positive population, increasing from 16% in the immunocompetent to 22% in the NK-depleted CTL condition (Fig. 5a). Also note that there is a strong induction in the number of CD11b$^{+}$GR-1$^{-}$ cells in the NK-deficient CTL culture, confirming the *ex vivo* FACS results (Fig. 3iii).

These data further strengthen the notion that α-ASGM-1 treatment did not deplete the activated T cell pool, but rather indicate that NK-depletion alters the functional phenotype of the myeloid splenic fraction to a more suppressive one. However, note that removal of the adherent fraction did not bring the lytic potential of the NK-depleted CTL culture to the same level as the immunocompetent CTLs, suggesting that some of the effects of *in vivo* NK depletion cannot be reverted by removing suppressive macrophages during *in vitro* restimulation. One possibility may reside in the effect of NK depletion on the dendritic cell pool. Indeed, the *ex vivo* results represented in Fig. 3 suggest that the CD11c$^{high}$ expressing DC pool is underrepresented in NK-depleted cultures, which could result in inefficient

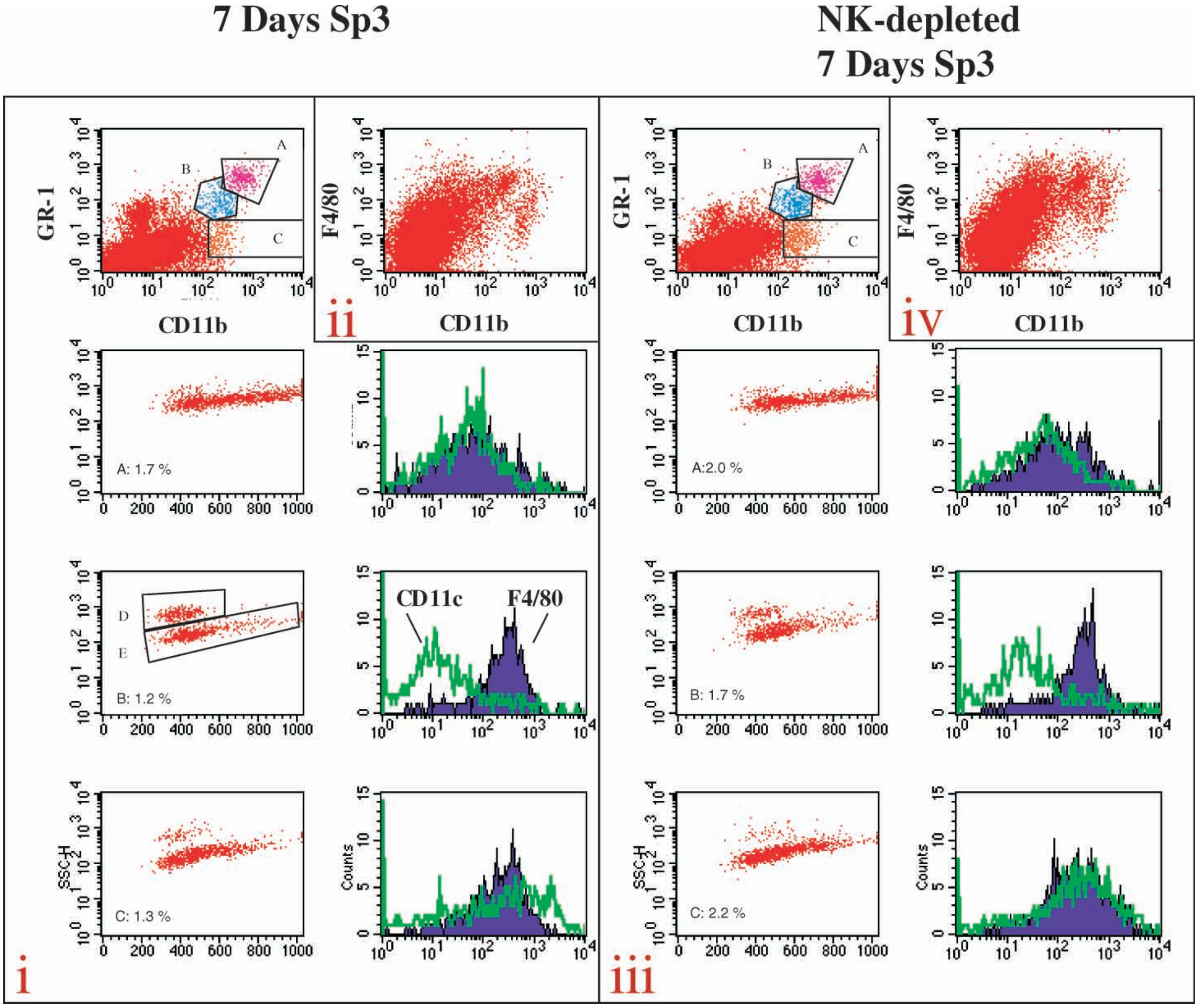

FIGURE 3 CD11b$^+$/GR-1$^+$ cells are enriched and underwent a phenotypical shift in NK-depleted tumor bearing mice. Splenocytes from immunocompetent and α-ASGM-1-treated, day 7, tumor-bearing mice were collected and after Fc receptor blocking immediately surface-stained with myeloid-specific antibodies. Splenocytes were double-stained with α-GR-1-PE and α-CD11b-FITC and subsequently subdivided in CD11b$^-$, CD11b$^{high}$GR-1$^{high}$ (population A), CD11b$^+$GR-1$^+$ (population B) and CD11b$^+$GR-1$^{low}$ (population C) populations for immunocompetent (i) and NK-depleted (iii) conditions. SSC/FSC profiles and histograms representing the distribution of F4/80 and CD11c expression are represented for each of the CD11b$^+$ subpopulations. Population B was further subdivided in two cell groups based on their SSC/FSC profiles (groups D and E). Macrophage maturation stage in the spleen was further determined by plotting the expression of F4/80 in function of CD11b for as well immunocompetent (ii) as NK depleted (iv) recipients.

antigen presentation in the splenic compartment during tumor challenge. Accordingly, recent results from Piccioli *et al.*, reported on stimulatory effects of NK on autologous DC maturation and DC cytokine production (Piccioli *et al.*, 2002).

Since NK cells can be active producers of cytokines, supporting as well type 1 as type 2 immune responses (Cooper *et al.*, 2001; Loza and Perussia, 2001; Deniz *et al.*, 2002), we also checked the cytokine profiles in the spleen 72 h after initiation of *in vitro* restimulation. As expected, effective induction of CTL activity was paralleled by high levels of IFN-γ secretion during *in vitro* culture (Fig. 5B). Depletion of NK cells not only abrogated IFN-γ production, but also significantly induced the production of type 2-associated cytokines IL-4, IL-13 and IL-10 (Fig. 5B). This implies that in our AKR derived BW-Sp3 tumor model, NK cells are prerequisite for the generation of type 1 immune responses and that in their absence, the immune system will have a tendency to further evolve in the direction of type 2-dominated responses. To analyze the implication of the shift in the type1/type2 cytokine balance on the activation status of the myeloid population, we also measured NO production in the cultures and arginase activity in lysates of the adherent fractions (Fig. 5B). Interestingly, macrophages that were activated in immunocompetent conditions tend to produce NO (classical activation), while macrophages activated in the absence of NK primordially produce L-ornithine and urea via the arginase pathway (alternative activation) (Munder *et al.*, 1998). Note also that the production of monocyte chemoattractant protein-1 (MCP-1), possibly implicated in the recruitment of myeloid cells to the spleen, is

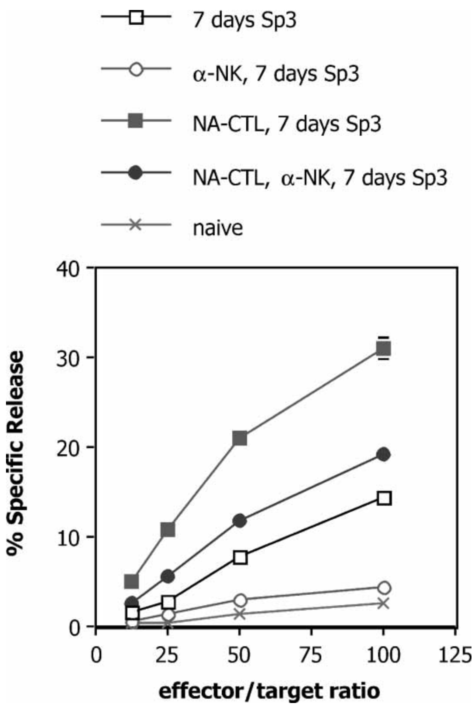

FIGURE 4 Functional role of NK cells in generation of anti-BW-Sp3 CTLs. Splenocytes from naïve, immunocompetent or α-ASGM-1-treated, 7 days, BW-Sp3 tumor bearing AKR mice were restimulated *in vitro* with irradiated cancer cells. After 5 days, viable lymphocytes were collected, (open symbols). Alternatively, 2 days after initiation of restimulation, adherent splenocytes were removed and restimulation of the non-adherent fraction was continued for another 3 days to obtain so-called non-adherent effectors (NA-CTL) (filled symbols). Cytolytic activity of effector CTL was measured in a 4 h $^{111}$In-release assay against BW-Sp3(B7-1) target cells.

reduced in immunocompetent CTL cultures (Legdeur *et al.*, 2001). Altogether our results indicate that NK depletion can selectively fuel the development of type 2-immune responses.

Our results complement some of the observations made with immunosuppressive macrophages that have been identified in the lymphoid organs of immunosuppressed, tumor-bearing mice and comprise a population of immature Gr1$^+$/CD11b$^+$ myeloid precursors (Bronte *et al.*, 1999; Kusmartsev *et al.*, 2000; Gabrilovich *et al.*, 2001). Exposure of these Gr1$^+$/CD11b$^+$ cells to IL-4 (type 2 cytokine) greatly increased the T lymphocyte suppressive activity of this population, while type 1 cytokines induced their maturation into competent mature DC1 (Apolloni *et al.*, 2000; Bronte *et al.*, 2000). Besides those functional similarities, the myeloid suppressor cells show some important differences from alternatively activated macrophages described by us and others. The authors themselves acknowledge the dissimilarities between their Gr-1$^+$/CD11b$^+$ adherent population and M2, stressing the immature phenotype of their suppressor myeloid cells (Bronte *et al.*, 2000). Moreover, we can add some additional deviating features: First, none of the studies report the production of arginase in their suppressor cells. Finally, the alternatively activated macrophages in our model express Fas-L, FcRγ and B7 (unpublished results) in contrast to the myeloid precursor (Bronte *et al.*, 2000). Further characterization of the phenotype of the suppressor cells and mechanism of suppression is currently under investigation.

### IFN-γ can Boost CTL Activity in NK-competent and Depleted Conditions

IFN-γ is one of the main factors produced by NK cells, early after immune challenge with cancer cells or infectious agents. Moreover NK-produced IFN-γ has been described to be critical for the promotion of adaptive T cell responses (Seaman *et al.*, 1987; Gray *et al.*, 1994; Salazar-Mather *et al.*, 1996, 1998; Smyth and Kelly, 1999). Since in our experimental set-up, NK depletion parallels the lack of IFN-γ production, we decided to determine the relative importance of IFN-γ for the induction of antitumor CTLs. To this end, we provided recombinant IFN-γ to immunocompetent or NK-depleted tumorbearing mice on one hand and blocking α-IFN-γ antibodies to immunocompetent BW-Sp3 inoculated mice on the other hand. Provision of anti-IFN-γ during tumor growth reduced the CTL-inducing capacity of tumor-challenged mice. Indeed, the lytic activity of CTLs derived from anti-IFN-γ-treated mice (6.6% specific release at effector/target ratio 100/1) barely exceeds the lytic activity of NK-deficient CTL cultures (4.5% specific release) (Fig. 6A). Providing excess IFN-γ to α-ASGM-1 treated mice, brings the efficiency of CTL induction almost to the level of immunocompetent mice (12% specific release in IFN-γ treated NK deficient mice, as compared to 15% in immunocompetent hosts, Fig. 5A). However, providing the same quantities of IFN-γ to immunocompetent recipients, results in an even more drastic increase in CTL activity. Percentage specific release goes from 15%, using CTLs from normal 7 day BW-Sp3 tumorbearers as effectors, to 47% specific release when IFN-γ is provided during tumor growth (Fig. 6A). This indicates that IFN-γ is very efficient at boosting early CTL responses, especially in immunocompetent, but only to a lesser extend in immunodysfunctional environments. We confirmed this observation by determining the influence of IFN-γ-treatment on the activation status of splenic macrophages in the CTL cultures. Though IFN-γ is typically used to steer macrophages to classical, type 1-dependent activation (Munder *et al.*, 1998), in our experimental set-up, IFN-γ will only have marginal effects on macrophage phenotype. Indeed, though arginase activity is reduced by IFN-γ (from 80 to 0 mU) and marginally induced by α-IFN-γ treatment (from 80 to 130 mU) in immunocompetent mice, provision of IFN-γ to NK-depleted mice will not reduce the inherent type 2 prevalence of the macrophage population. Thus NK-produced IFN-γ cannot solely explain the CTL-deficiency observed in NK-depleted mice, indicating that other NK factors and/or functions are involved in biasing type 2 responses in the spleen. A direct interaction between BW-Sp3 cancer cells and innate cells (NK cells, activated macrophages) via Rae-1/NKG2D

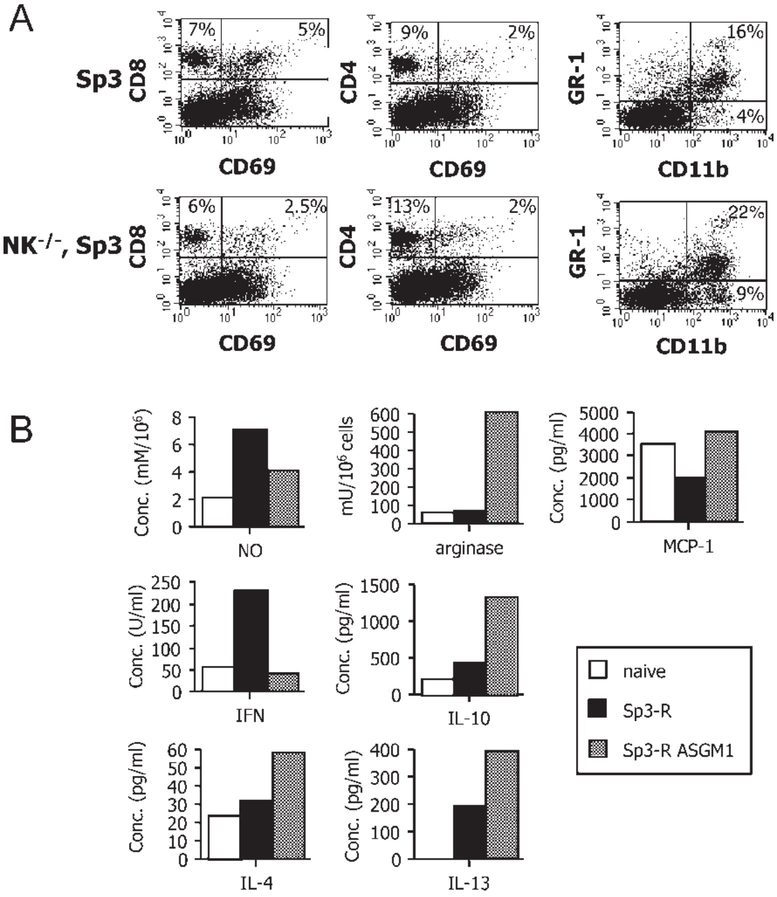

FIGURE 5 Depletion of NK cells alters the cellular composition and the Th1/Th2 cytokine balance in splenic derived CTL cultures. Splenocytes from naïve, immunocompetent or α-ASGM-1-treated, 7 days, BW-Sp3 tumor bearing AKR mice were restimulated *in vitro* with irradiated cancer cells. After 3 days, cell-free supernatants were collected, adherent cells were separated from non-adherent cells and subjected to analysis. (A) Percentages of $CD8^+/CD69^+$, $CD4^+/CD69^+$ T cells (non-adherent) and $GR\text{-}1^+/CD11b^+$ myeloid cells (adherent) in 3 day restimulated CTL cultures were analyzed by FACS and represented in Dot Plots. (B) Supernatant from CTL cultures was tested for NO (mM/$10^6$ cells), IFN-γ (U/ml), IL-4 (pg/ml), IL-10 (pg/ml), IL-13 (pg/ml) and MCP-1 (pg/ml). Arginase activity was determined in the adherent fraction of restimulated spleens. The concentrations are represented as quantity produced per $10^6$ adherent cells.

might be a primary stimulus that induces the cascade of cellular immune responses leading to type 1 associated adaptive tumor immunity (including generation of CTLs) (Cerwenka *et al.*, 2000, 2001; Diefenbach *et al.*, 2001). Consequently, in this scenario the link between CTL and NK activity could rely primarily on the production of NK-derived type 1 cytokines as was suggested in other studies (Kurosawa *et al.*, 1995; Smyth and Kelly, 1999). However, the evidence that was so far provided for a direct role of NK-derived IFN-γ and/or other cytokines on CTL generation is debatable (see "Introduction") and is further refuted by our results.

### Conditioning of Splenic Adherent Cells in a Dominant Type 2 Environment Induces their NK Susceptibility

Considering that APC can become sensitive targets for NK lysis if they express the proper balance of NK activatory/inhibitory signals (Gilbertson *et al.*, 1986; Djeu and Blanchard, 1988a,b; Chambers *et al.*, 1996; Geldhof *et al.*, 1998a; Carbone *et al.*, 1999; Parajuli *et al.*, 1999; Warschkau and Kiderlen, 1999; Wilson *et al.*, 1999a,b; Spaggiari *et al.*, 2001) and taking into account that NK-produced IFN-γ is not sufficient to revert the Type 2 propensity of macrophages (Fig. 6B), we considered the possibility that the lytic function of NK cells may contribute to its adverse effect on Type 2 macrophages. Therefore, we collected the adherent fractions of immunocompetent and NK-depleted CTL cultures, 3 days after initiation of the *in vitro* cultures and included them as targets in a LAK assay. Using IL-12 activated NK cells as effectors we could clearly detect superior lysis of Type 2 macrophages as compared to Type 1 conditioned macrophages (Fig. 7). Notably, adherent cells isolated from naïve splenic cultures showed intermediate sensitivity, suggesting that Type 1-dependent activation will protect macrophages from NK-mediated lysis, while Type 2 activation will induce their NK sensitivity (Fig. 7). Thus, alternatively activated macrophages from NK-deficient conditions were significantly better lysed than classically activated macrophages from immunocompetent cultures. This is consistent with early publications suggesting that *in vitro* IFN-γ (Type 1 cytokine) treatment of tumor cells (Gronberg *et al.*, 1989) or monocytes (Djeu and Blanchard, 1988a,b) can confer protection to LAK lysis. Hence NK cells can be involved in preferential

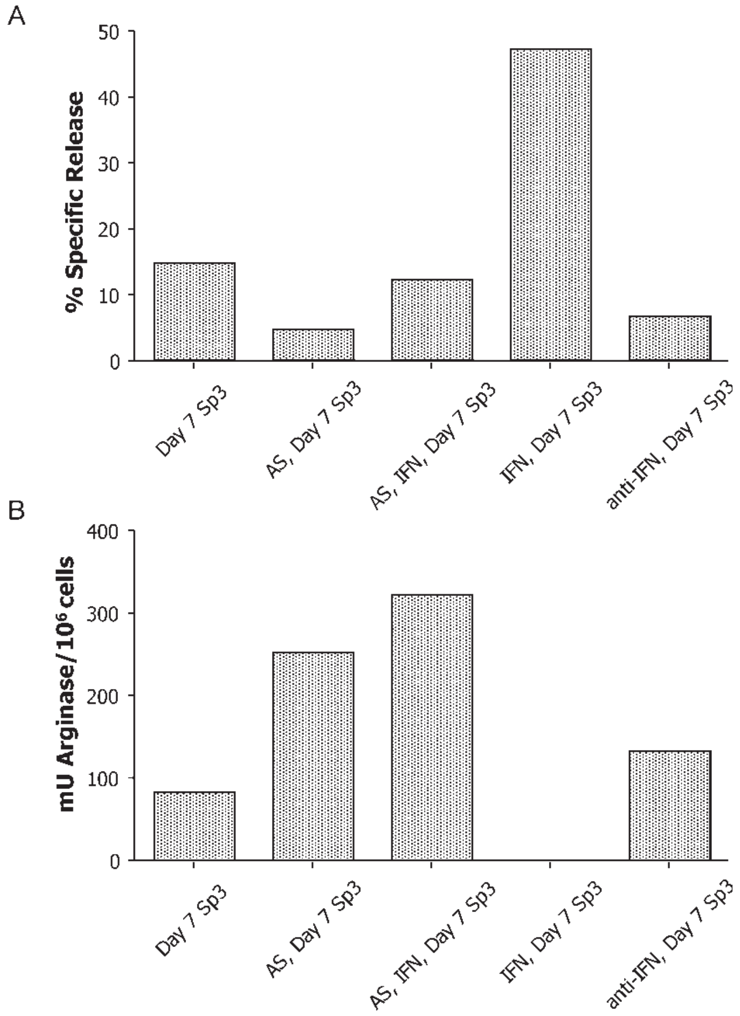

FIGURE 6 IFN-γ can boost overall CTL responses but does not block the development of arginase-producing alternatively activated macrophages. Mice were treated with α-IFN-γ, α-ASGM-1 and/or recombinant IFN-γ and subsequently challenged with BW-Sp3 subcutaneously. Seven days after tumor inoculation, spleens were harvested and restimulated in the presence of irradiated BW-Sp3(B7-1) for 5 days. (A) Viable non-adherent splenocytes were collected and included in an *in vitro* cytotoxicity assay. The results represent cytolytic activity toward BW-Sp3(B7-1) at an E/T ratio of 100/1. (B) The adherent fraction of the CTL cultures were collected by scraping and arginase activity was determined in the lysate of $10^6$ viable cells.

physical elimination of alternatively activated macrophages *in vivo*.

In conclusion, the herein presented results unequivocally link NK activity and CTL-mediated anti-tumor responses and furthermore provide evidence for novel regulatory mechanisms possibly implicated in the NK/CTL interaction. Indeed, our analysis of the involvement of NK cells in the BW-Sp3 tumor model revealed the following main findings: (i) Progression of BW-Sp3 tumors leads to impaired anti-cancer cell CTL and NK cell activities and depletion of NK cells during the early phase of BW-Sp3 tumor development impairs the development of CTL responses and increases tumor progression; (ii) NK cell depletion during early BW-Sp3 tumor formation changes the macrophage number and phenotype in the spleen, leading to increased levels of $CD11b^+/GR\text{-}1^+$ double positive cells; (iii) NK-depletion alters the tumor-elicited splenic cytokine production in favor of a Type 2 cytokine microenvironment and promotes the development of alternatively activated splenic macrophages; (iv) M2 enriched splenic adherent cells suppress potently CTL activity; (v) provision of excess IFN-γ can boost immunocompetent and NK-depleted CTL activity, but does not revert underlying NK-mediated macrophage defects; (vi) alternatively activated splenic macrophages cells are more susceptible to NK-mediated lysis as compared to classically activated macrophages.

Collectively, we provide evidence for the involvement of NK/macrophage lytic interactions in the switch from Th2 to Th1-dependent immune responses and corroborate the crucial role of NK cells in the development and maintenance of anti-tumor CTLs. Our study further points to a new regulatory mechanism possibly implicated in the NK/CTL interaction, namely NK-mediated control of suppressive M2-like populations, that may rely on both NK-mediated regulatory (i.e., initiation of type 1 oriented signaling) as well as NK-mediated cytolytic (i.e., elimination of M2 cells) activities. Hence a regulatory pathway linking NK cells, M2 cells and CTLs can be operative in cancer.

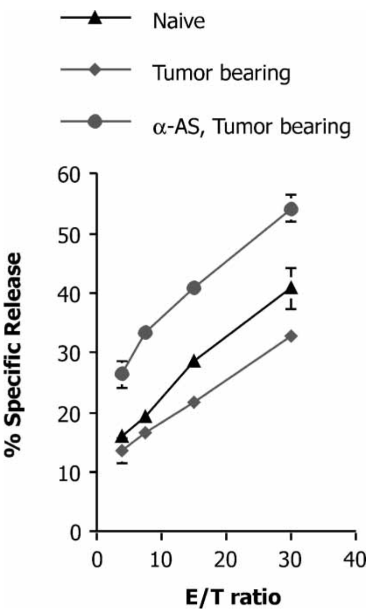

FIGURE 7 LAK cells differentially lyse M1 vs. M2 cells. The activity of IL-2/IL-12 activated LAK cells was measured in an *in vitro* cytotoxicity assay. Sensitivity of adherent cells, isolated from spleen cells cultured during 3 days *in vitro*, to LAK effector cells was tested. Spleens were isolated from naïve (▲), 7 day BW-Sp3 tumor bearing (◆), and α-ASGM-1-treated, 7 day BW-Sp3 tumor bearing (•) AKR. Spontaneous release was ≤ 17% for splenocytes. One representative experiment of two is shown, indicating the mean percentage specific release of targets in triplicate (± s.e.m.). Standard deviations of less than 3% are not shown for sake of clarity.

### MATERIALS AND METHODS

### Mice

Specific pathogen-free female AKR (Thy1.1, H-$2^k$) mice were obtained from Harlan CPB (Zeist, The Netherlands).

### Tumor Cell Lines

The BW-Sp3 cell line was derived from the original spontaneous BW5147 T cell lymphoma (referred to as BW-O) (AKR origin, Salk Institute, La Jolla, CA) by *in vitro* and *in vivo* passages, as described previously (Geldhof *et al.*, 1998b). The generation of BW-Sp3(B7-1) was described earlier (Raes *et al.*, 1998).

### CTL Induction

For the generation of CTL, spleens from tumor-bearing mice were taken and splenocytes from two spleens were restimulated in flasks in the presence of $10^7$ irradiated (110 Gy) cancer cells. Five days later the cytolytic activity of the viable lymphocytes, separated on a Ficoll gradient (Amersham Pharmacia), were tested in a classical $^{111}$In release assay (Geldhof *et al.*, 1995). Alternatively, adherent cells from primary CTL cultures were removed by transferring the non-adherent fractions to new plates, 2 days after initiation of the CTL restimulation and culture was continued for another 3 days before they were included in CTL assays.

### *In Vivo* Experimental Settings

For *in vivo* NK depletion, AKR mice were injected intravenously in the tail with 0.2 ml of a tenfold dilution in phosphate buffered saline (PBS) of α-ASGM-1 antibodies (Wako Chemicals, Osaka, Japan), 24 h prior to tumor inoculation and repeated every 4–5 days during the course of the experiment. In NK depletion experiments, specific depletion was greater than 95%. Mice in groups of six were injected subcutaneously in the right flank with $2.10^6$ cancer cells in 0.2 ml PBS. For every treatment, mortality of the hosts, fraction of regressors/progressors and tumor growth (by measuring the local tumor diameter) were followed up twice a week. For *in vivo* treatment with recombinant IFN-γ, mice were injected intraperitoneally with 100.000 units IFN-γ 24 h prior to and 3 days after tumor injection. Treatment with α-IFN-γ antibodies was done once, injecting 0.5 mg intraperitoneally 24 h before tumor treatment.

### Generation of A-LAK Cells

Mouse spleen cells were isolated and nylon wool non-adherent cells were harvested as described in detail elsewhere (Geldhof *et al.*, 1995, 1998a). The nylon wool non-adherent cells were brought to a concentration of $2 \times 10^6$/ml in RPMI medium, supplemented with 0.3 mg/ml L-glutamine, 100 U/ml penicillin, 0.1 mg/ml streptomycin, 10% FCS, $5 \times 10^{-5}$ M mercapto-ethanol ( = CM) and 1000 U/ml IL-2 (Eurocetus, Netherlands) and incubated at 37°C in 5% $CO_2$ and 100% humidity during 5 days. For the generation of IL-2/IL-12 LAK, recombinant mouse IL-12 (generously provided by Genetics Institute, Inc., Cambridge, MS, USA), was added to the IL-2 LAK cultures at 100 U/ml, 72 h after initiation of the cultures for another 48 h. The adherent cell fraction is harvested with 0.01% EDTA in PBS and used as effector cell population in $^{111}$In-release cytotoxicity assays.

### $^{111}$In-release Cytotoxicity Assay

The target cells were labeled with $^{111}$In by incubating $10^6$ cells in 100 μl (RPMI + 5% FCS) with 0.1 mCi $^{111}$InCl (Amersham, Belgium) during 10 min (Geldhof *et al.*, 1995). After extensive washing, $10^4$ target cells were incubated with 2-fold dilutions of effector cells in a total volume of 200 μl CM in 96-well round bottom plates. All experiments were performed in triplicates and twice repeated. After 4 h at 37°C, the plates are centrifuged 3 min at 600 r.p.m., 100 μl of supernatants is collected and radiation is counted in a γ-counter. The percentage of specific lysis was calculated as ((experimental release − spontaneous release)/(maximal release − spontaneous release)) × 100, where the spontaneous release was determined from labeled targets cells incubated without effector cells and maximal lysis from target cells incubated 1 h before harvesting in 2% SDS.

### Determination of Arginase Activity

Arginase activity was measured in cell lysates with slight modifications as previously described (Namangala *et al.*, 2001). Briefly, cells were lysed with 100 μl of 0.1% Triton X-100. After 30 min on a shaker, 100 μl of 25 mM Tris-HCl was added. To 100 μl of this lysate, 35 μl of 10 mM $MnCl_2$ was added, and the enzyme was activated by heating for 10 min at 56°C. Arginine hydrolysis was conducted by incubating 40 μl lysate with 40 μl of 0.5 M L-arginine, pH 9.7, at 37°C for 60 min. The reaction was stopped with 320 μl of $H_2SO_4/H_3PO_4/H_2O$ (1/3/7, v/v/v). The urea concentration was measured at 540 nm after addition of 16 μl of 9% α-isonitrosopropiophenone (dissolved in 100% ethanol) followed by heating at 95°C for 30 min. One unit of enzyme activity is defined as the amount of enzyme that catalyzes the formation of 1 μmol of urea per minute.

### NO Measurement

NO was measured as nitrite using the Griess reagent. Culture supernatant was mixed with 100 μl of 1% sulfanilamide, 0.1% N-(1-naphthyl)ethylenediamine dihydrochloride, and 2.5% $H_3PO_4$. Absorbance was measured at 540 nm in a microplate reader.

### Cytokine Assays

The production of IL-4, IL-10, IL-13, MCP-1, and IFN-γ was quantified by subjecting culture supernatants to commercially available (Pharmingen, San Diego, CA, USA) sandwich ELISA tests according to the manufacturers' protocols.

### FACS Staining and Analysis of Adherent and Non-Adherent Spleen Cells

Cell samples comprising $10^6$ cells were incubated with the appropriate dilutions of antibodies, as suggested by the distributors, at 4°C for periods of 30 min. Rat anti-CD11b-FITC, anti-CD69-FITC, anti-CD25-FITC, anti-GR-1-PE, anti-CD4-PE, anti-CD8-PE, anti-CD11c-biotin and SA-APC were purchased from Pharmingen (San Diego, CA, USA), anti-F4/80-biotin from Serotec. To prevent FcR-mediated binding of staining antibodies, the Fc-receptors were blocked with 2.4G2 (ATTC) derived $F(ab)_2$ antibodies, prior to the addition of the indicated antibodies. As isotype controls for a specific binding of rat, mouse and hamster anti-mouse antibodies, control antibodies were purchased from the distributors and included during FACS staining. The stained cells were subjected to FACS analysis with the Becton-Dickinson FACS Vantage coupled to an Apple Macintosh FACStation. The results were analyzed with the CellQuest program.

### *Acknowledgements*

This research was supported by grants from the Foundation of Scientific Research-Flanders (FWO-no. 1.5.213.00). A.B.G. is a Post-doctoral Fellow of the Foundation of Scientific Research-Flanders (FWO). We would like to thank L. Brijs, M. Gobert, E. Vercauteren and E. Omasta for an excellent technical assistance, K. Van Beneden for providing anti-NKG2D antibodies and both Hoffman-LaRoche, Inc. and Genetics Institute, Inc. for supplying the recombinant IL-12 used for this work.

### *References*

Apolloni, E., Bronte, V., Mazzoni, A., Serafini, P., Cabrelle, A., Segal, D.M., Young, H.A. and Zanovello, P. (2000) "Immortalized myeloid suppressor cells trigger apoptosis in antigen-activated T lymphocytes", *J. Immunol.* **165**(12), 6723–6730.

Beck, B.N., Gillis, S. and Henney, C.S. (1982) "Display of the neutral glycolipid ganglio-n-tetraosylceramide (asialo GM1) on cells of the natural killer and T lineages", *Transplantation* **33**, 118–122.

Bronte, V., Chappell, D.B., Apolloni, E., Cabrelle, A., Wang, M., Hwu, P. and Restifo, N.P. (1999) "Unopposed production of granulocyte-macrophage colony-stimulating factor by tumors inhibits CD8+ T cell responses by dysregulating antigen-presenting cell maturation", *J. Immunol.* **162**, 5728–5737.

Bronte, V., Apolloni, E., Cabrelle, A., Ronca, R., Serafini, P., Zamboni, P., Restifo, N.P. and Zanovello, P. (2000) "Identification of a CD11b(+)/Gr-1(+)/CD31(+) myeloid progenitor capable of activating or suppressing CD8(+) T cells", *Blood* **96**(12), 3838–3846.

Bronte, V., Serafini, P., Apolloni, E. and Zanovello, P. (2001) "Tumor-induced immune dysfunctions caused by myeloid suppressor cells", *J. Immunother.* **24**(6), 431–446.

Carbone, E., Terrazzano, G., Ruggiero, G., Zanzi, D., Ottaiano, A., Manzo, C., Karre, K. and Zappacosta, S. (1999) "Recognition of autologous dendritic cells by human NK cells", *Eur. J. Immunol.* **29**, 4022–4029.

Carbone, E., Terrazzano, G., Melian, A., Zanzi, D., Moretta, L., Porcelli, S., Karre, K. and Zappacosta, S. (2000) "Inhibition of human NK cell-mediated killing by CD1 molecules", *J. Immunol.* **164**, 6130–6137.

Cerwenka, A., Bakker, A.B., McClanahan, T., Wagner, J., Wu, J., Phillips, J.H. and Lanier, L.L. (2000) "Retinoic acid early inducible genes define a ligand family for the activating NKG2D receptor in mice", *Immunity* **12**, 721–727.

Cerwenka, A., Baron, J.L. and Lanier, L.L. (2001) "Ectopic expression of retinoic acid early inducible-1 gene (RAE-1) permits natural killer cell-mediated rejection of a MHC class I-bearing tumor *in vivo*", *Proc. Natl. Acad. Sci. USA* **98**, 11521–11526.

Chambers, B.J., Salcedo, M. and Ljunggren, H.G. (1996) "Triggering of natural killer cells by the costimulatory molecule CD80 (B7-1)", *Immunity* **5**, 311–317.

Cooper, M.A., Fehniger, T.A., Turner, S.C., Chen, K.S., Ghaheri, B.A., Ghayur, T., Carson, W.E. and Caligiuri, M.A. (2001) "Human natural killer cells: a unique innate immunoregulatory role for the CD56(bright) subset", *Blood* **97**, 3146–3151.

Deniz, G., Akdis, M., Aktas, E., Blaser, K. and Akdis, C.A. (2002) "Human NK1 and NK2 subsets determined by purification of IFN-gamma-secreting and IFN-gamma-nonsecreting NK cells", *Eur. J. Immunol.* **32**(3), 879–884.

Diefenbach, A., Jensen, E.R., Jamieson, A.M. and Raulet, D.H. (2001) "Rae1 and H60 ligands of the NKG2D receptor stimulate tumour immunity", *Nature* **413**, 165–171.

Djeu, J.Y. and Blanchard, D.K. (1988a) "Lysis of human monocytes by lymphokine-activated killer cells", *Cell Immunol.* **111**, 55–65.

Djeu, J.Y. and Blanchard, D.K. (1988b) "Interferon-gamma-induced alterations of monocyte susceptibility to lysis by autologous lymphokine-activated killer (LAK) cells", *Int. J. Cancer* **42**(3), 449–454.

Ferlazzo, G., Semino, C. and Melioli, G. (2001) "HLA class I molecule expression is up-regulated during maturation of dendritic cells, protecting them from natural killer cell-mediated lysis", *Immunol. Lett.* **76**, 37–41.

Gabrilovich, D.I., Velders, M.P., Sotomayor, E.M. and Kast, W.M. (2001) "Mechanism of immune dysfunction in cancer mediated by immature Gr-1+ myeloid cells", *J. Immunol.* **166**, 5398–5406.

Geldhof, A.B., Raes, G., Bakkus, M., Devos, S., Thielemans, K. and De Baetselier, P. (1995) "Expression of B7-1 by highly metastatic mouse T lymphomas induces optimal natural killer cell-mediated cytotoxicity", *Cancer Res.* **55**, 2730–2733.

Geldhof, A.B., Moser, M., Lespagnard, L., Thielemans, K. and de Baetselier, P. (1998a) "IL-12-Activated NK cells recognize B7 costimulatory molecules on tumor cells and autologous dendritic cells", *Blood* **91**, 196–206.

Geldhof, A.B., van Ginderachter, J., Van der Steen, P., Raes, G. and de Baetselier, P. (1998b) "Multiple effects of transfection with interleukin 2 and/or interferon gamma on the behavior of mouse T lymphoma cells", *Clin. Exp. Metastasis* **16**, 447–459.

Gilbertson, S.M., Shah, P.D. and Rowley, D.A. (1986) "NK cells suppress the generation of Lyt-2+ cytolytic T cells by suppressing or eliminating dendritic cells", *J. Immunol.* **136**, 3567–3571.

Goerdt, S. and Orfanos, C.E. (1999) "Other functions, other genes: alternative activation of antigen-presenting cells", *Immunity* **10**, 137–142.

Goerdt, S., Politz, O., Schledzewski, K., Birk, R., Gratchev, A., Guillot, P., Hakiy, N., Klemke, C.D., Dippel, E., Kodelja, V. and Orfanos, C.E. (1999) "Alternative versus classical activation of macrophages", *Pathobiology* **67**, 222–226.

Gray, J.G., Hirokawa, M. and Horwitz, D.A. (1994) "The role of transforming growth factor in the generation of suppression: an interaction between CD8+ T and NK cells", *J. Exp. Med.* **180**, 1937–1942.

Gronberg, A., Ferm, M., Tsai, L. and Kiessling, R. (1989) "Interferon is able to reduce tumor cell susceptibility to human lymphokine-activated killer (LAK) cells", *Cell Immunol.* **118**(1), 10–21.

Kos, F.J. and Engleman, E.G. (1996) "Role of natural killer cells in the generation of influenza virus-specific cytotoxic T cells", *Cell Immunol.* **173**, 1–6.

Kurosawa, S., Harada, M., Matsuzaki, G., Shinomiya, Y., Terao, H., Kobayashi, N. and Nomoto, K. (1995) "Early-appearing tumour-infiltrating natural killer cells play a crucial role in the generation of anti-tumour T lymphocytes", *Immunology* **85**, 338–346.

Kusmartsev, S.A., Li, Y. and Chen, S.H. (2000) "Gr-1+ myeloid cells derived from tumor bearing mice inhibit primary T cell activation induced through CD3/CD28 costimulation", *J. Immunol.* **165**, 779–785.

Legdeur, M.C., Broekhoven, M.G., Schuurhuis, G.J., Beelen, R.H. and Ossenkoppele, G.J. (2001) "Monocyte-chemoattractant-protein-1-mediated migration of human monocytes towards blasts from patients with acute myeloid leukemia", *Cancer Immunol. Immunother.* **50**(1), 16–22.

Loza, M.J. and Perussia, B. (2001) "Final steps of natural killer cell maturation: a model for type 1-type 2 differentiation?", *Nat. Immunol.* **2**(10), 917–924.

MacDonald, A.S., Straw, A.D., Bauman, B. and Pearce, E.J. (2001) "CD8(−) dendritic cell activation status plays an integral role in influencing Th2 response development", *J. Immunol.* **167**, 1982–1988.

Mazzoni, A., Bronte, V., Visintin, A., Spitzer, J.H., Apolloni, E., Serafini, P., Zanovello, P. and Segal, D.M. (2002) "Myeloid suppressor lines inhibit T cell responses by an NO-dependent mechanism", *J. Immunol.* **168**(2), 689–695.

Munder, M., Eichmann, K. and Modolell, M. (1998) "Alternative metabolic states in murine macrophages reflected by the nitric oxide synthase/arginase balance: competitive regulation by CD4+ T cells correlates with Th1/Th2 phenotype", *J. Immunol.* **160**, 5347–5354.

Namangala, B., de Baetselier, P., Noël, W., Brijs, L. and Beschin, A. (2001) "Alternative versus classical macrophage activation during experimental African trypanosomosis", *J. Leukoc. Biol.* **69**, 387–396.

Parajuli, P., Nishioka, Y., Nishimura, N., Singh, S.M., Hanibuchi, M., Nokihara, H., Yanagawa, H. and Sone, S. (1999) "Cytolysis of human dendritic cells by autologous lymphokine-activated killer cells: participation of both T cells and NK cells in the killing", *J. Leukoc. Biol.* **65**, 764–770.

Parker, S.E., Sun, Y.H. and Sears, D.W. (1988) "Differential expression of the ASGM1 antigen on anti-reovirus and alloreactive cytotoxic T lymphocytes (CTL)", *J. Immunogenet.* **15**, 215–226.

Piccioli, D., Sbrana, S., Melandri, E. and Valiante, N.M. (2002) "Contact-dependent stimulation and inhibition of dendritic cells by natural killer cells", *J. Exp. Med.* **195**(3), 335–341.

Pulendran, B., Palucka, K. and Banchereau, J. (2001) "Sensing pathogens and tuning immune responses", *Science* **293**, 253–256.

Raes, G., Geldhof, A., Vanden Driessche, T., Opdenakker, G., Sibille, C. and De Baetselier, P. (1995) "Immunogenization of a murine T-cell lymphoma via transfection with interferon-gamma", *Leukemia* **9**, S121–S127.

Raes, G., Van Ginderachter, J., Liu, Y.-Q., Brys, L., Thielemans, K., De Baetselier, P. and Geldhof, A. (1998) "Active antitumor immunotherapy, with or without B7-mediated costimulation, increases tumor progression in an immunogenic murine T cell lymphoma model", *Cancer Immunol. Immunother.* **45**, 257–265.

Salazar-Mather, T.P., Ishikawa, R. and Biron, C.A. (1996) "NK cell trafficking and cytokine expression in splenic compartments after IFN induction and viral infection", *J. Immunol.* **157**, 3054–3064.

Salazar-Mather, T.P., Orange, J.S. and Biron, C.A. (1998) "Early murine cytomegalovirus (MCMV) infection induces liver natural killer (NK) cell inflammation and protection through macrophage inflammatory protein 1alpha (MIP-1alpha)-dependent pathways", *J. Exp. Med.* **187**, 11–14.

Seaman, W.E., Sleisenger, M., Eriksson, E. and Koo, G.C. (1987) "Depletion of natural killer cells in mice by monoclonal antibody to NK1.1: reduction in host defence against malignancy without loss of cellular or humoral immunity", *J. Immunol.* **138**, 4539–4544.

Slifka, M.K., Pagarigan, R.R. and Whitton, J.L. (2000) "NK markers are expressed on a high percentage of virus-specific CD8+ and CD4+ T cells", *J. Immunol.* **164**(4), 2009–2015.

Smyth, M.J. and Kelly, J.M. (1999) "Accessory function for NK1.1+ natural killer cells producing interferon-gamma in xenospecific cytotoxic T lymphocyte differentiation", *Transplantation* **68**, 840–843.

Smyth, M.J., Kershaw, M.H. and Darcy, P.K. (1998) "Xenospecific CD8+ cytotoxic T lymphocyte generation: accessory function for CD4+ T cells and natural killer 1.1+ cells", *Transplantation* **65**, 1278–1281.

Spaggiari, G.M., Carosio, R., Pende, D., Marcenaro, S., Rivera, P., Zocchi, M.R., Moretta, L. and Poggi, A. (2001) "NK cell-mediated lysis of autologous antigen-presenting cells is triggered by the engagement of the phosphatidylinositol 3-kinase upon ligation of the natural cytotoxicity receptors NKp30 and NKp46", *Eur. J. Immunol.* **31**, 1656–1665.

Stein, M., Keshav, S., Harris, N. and Gordon, S. (1992) "Interleukin 4 potently enhances murine macrophage mannose receptor activity: a marker of alternative immunologic macrophage activation", *J. Exp. Med.* **176**, 287–292.

Stitz, L., Baenziger, J., Pircher, H., Hengartner, H. and Zinkernagel, R.M. (1986) "Effect of rabbit anti-asialo GM1 treatment *in vivo* or with anti-asialo GM1 plus complement *in vitro* on cytotoxic T cell activities", *J. Immunol.* **136**, 4674–4680.

Stout, R.D., Schwarting, G.A. and Suttles, J. (1987) "Evidence that expression of asialo-GM1 may be associated with cell activation. Correlation of asialo-GM1 expression with increased total cellular RNA and protein content in normal thymocyte and spleen cell populations", *J. Immunol.* **139**, 2123–2129.

Terao, H., Harada, M., Kurosawa, S., Shinomiya, Y., Ito, O., Tamada, K., Takenoyama, M. and Nomoto, K. (1996) "The opposite effect of tumor-infiltrating natural killer cells on *in vivo* priming of tumor-specific CD8+ T cells and CD4+ T cells", *Immunobiology* **195**, 172–186.

Van den Driessche, T., Bakkus, M., Toussaint-Demylle, D., Thielemans, K., Verschueren, H. and de Baetselier, P. (1994) "Tumorigenicity of mouse T lymphoma cells is controlled by the level of major histocompatibility complex class I H-2$K^k$ antigens", *Clin. Exp. Metastasis* **12**, 73–83.

Warschkau, H. and Kiderlen, A.F. (1999) "A monoclonal antibody directed against the murine macrophage surface molecule F4/80 modulates natural immune response to Listeria monocytogenes", *J. Immunol.* **163**, 3409–3416.

Wilson, J.L., Charo, J., Martin-Fontecha, A., Dellabona, P., Casorati, G., Chambers, B.J., Kiessling, R., Bejarano, M.T. and Ljunggren, H.G. (1999a) "NK cell triggering by the human costimulatory molecules CD80 and CD86", *J. Immunol.* **163**, 4207–4212.

Wilson, J.L., Heffler, L.C., Charo, J., Scheynius, A., Bejarano, M.T. and Ljunggren, H.G. (1999b) "Targeting of human dendritic cells by autologous NK cells", *J. Immunol.* **163**, 6365–6370.

